# Genome-wide (ChIP-seq) identification of target genes regulated by BdbZIP10 during paraquat-induced oxidative stress

**DOI:** 10.1186/s12870-018-1275-8

**Published:** 2018-04-10

**Authors:** Ruth C. Martin, Kelly Vining, James E. Dombrowski

**Affiliations:** 1 USDA ARS National Forage Seed and Cereal Research Unit, 3450 SW Campus Way, Corvallis, OR 97330 USA; 2 Department of Horticulture, 4123 Agricultural & Life Sciences, Oregon State University, Corvallis, OR 97330 USA

**Keywords:** bZIP transcription factors, Oxidative stress, Zinc, Chromatin immunoprecipitation (ChIP), *Brachypodium*

## Abstract

**Background:**

bZIP transcription factors play a significant role in many aspects of plant growth and development and also play critical regulatory roles during plant responses to various stresses. Overexpression of the *Brachypodium bZIP10* (Bradi1g30140) transcription factor conferred enhanced oxidative stress tolerance and increased viability when plants or cells were exposed to the herbicide paraquat. To gain a better understanding of genes involved in bZIP10 conferred oxidative stress tolerance, chromatin immunoprecipitation followed by high throughput sequencing (ChIP-Seq) was performed on *BdbZIP10* overexpressing plants in the presence of oxidative stress.

**Results:**

We identified a transcription factor binding motif, TGDCGACA, different from most known bZIP TF motifs but with strong homology to the *Arabidopsis* zinc deficiency response element. Analysis of the immunoprecipitated sequences revealed an enrichment of gene ontology groups with metal ion transmembrane transporter, transferase, catalytic and binding activities. Functional categories including kinases and phosphotransferases, cation/ion transmembrane transporters, transferases (phosphorus-containing and glycosyl groups), and some nucleoside/nucleotide binding activities were also enriched.

**Conclusions:**

*Brachypodium* bZIP10 is involved in zinc homeostasis, as it relates to oxidative stress.

**Electronic supplementary material:**

The online version of this article (10.1186/s12870-018-1275-8) contains supplementary material, which is available to authorized users.

## Background

Transcription factors (TFs) are integral for the regulation of gene expression during the normal growth and development of all organisms and for facilitating responses to environmental and physiological stresses. As organisms become more complex, different families of TFs may increase in number and diversify in function [1]. This is evident in plant TFs such as the Dof (DNA-binding one finger) gene family [2], MADs-Box gene family [3] and the bZIP gene family in plants [4]. These bZIP TF families contain groups with members that control networks of genes that may be distinct from those controlled by their ancestral TF gene [5].

Within the plant kingdom, bZIP TFs are proposed to have evolved from four founder genes that were involved in oxidative stress and the unfolded protein response [5]. As plants have evolved, the number of bZIP TFs has increased, and their roles have expanded to include many aspects of plant growth including embryo development, germination, shoot development, flowering, and seed development [6]. The bZIP TFs also play critical regulatory roles during plant responses to various stresses including cold, drought, salt, heat, and submergence [5, 7].

The genomes of many plant species have been mined for genes encoding bZIP proteins, and the number of these proteins varies widely in different species, from 75 in *Arabidopsis* [8], 55 in *Vitis vinifera* L. [9], 131 in *Glycine max* (L.) Merr. [10], 272 in *Gossypium raimondii* Ulbr., and 95 in *Brachypodium distachyon* [11]. The four progenitor groups of bZIP proteins have evolved and are subdivided into 13 groups (A-L and S) based on their bZIP domains, conserved motifs, and the presence of introns in the coding region for the conserved basic motif of many angiosperms [5]. While the total number of bZIP proteins varies in different species, all plants have representatives within each of the 13 groups. Within each group, the possible groups of orthologous members have diverged from the same ancestral gene and usually are associated with similar processes; for example within Group A, many of the TFs are involved in abscisic acid related processes, many Group G members are involved in light responses and photomorphogenesis, Group C and S members tend to function in energy metabolism and oxidative stress responses, Groups D and E are involved in hormonal responses and plant development, and Groups K and B may be involved in the unfolded protein response [5]. Less is known about Groups F, J and L. Members of Group F-subgroup 1 are widely conserved across land plants [12], and two members of this subgroup, AtZIP19 and 23, have been characterized as zinc deficiency TFs [13]. More recently, the *Atbzip19* single mutant was shown to be unable to adapt to zinc deficiency conditions, while the *Atbzip23* single mutant was not severely affected under the same conditions [14]. Group F-subgroup 2 contains AtbZIP24, which is involved in abiotic stress responses [15], and is more variable across land plants [12].

Zinc deficiency was previously shown to induce oxidative stress related genes in wheat [16]. Furthermore, the BdbZIP10 (Bradi1g30140) TF, when overexpressed in *Brachypodium*, conferred enhanced oxidative stress resistance and increased viability when plants or cells were exposed to N,N′-dimethyl-4,4′-bipyridinium dichloride (common name: paraquat) [17], an herbicide which induces oxidative stress in plants [18, 19]. This BdbZIP10 TF is the homolog of *Arabidopsis* bZIP19 and *Arabidopsis* bZIP23 TFs, which have been characterized for their role in adaptation to zinc deficiency in *Arabidopsis*. Since overexpression of *BdbZIP10* increases oxidative stress tolerance [17], we investigated which genes this TF interacts with during the oxidative stress tolerance response. Chromatin immunoprecipitation followed by high throughput sequencing (ChIP-Seq) is valuable for identifying DNA binding sites of transcription factors [20, 21]. Paraquat-treated *Brachypodium* overexpressing the *BdbZIP10-GFP* fusion gene was used for chromatin immunoprecipitation using a GFP antibody that recognizes the BdbZIP10-GFP fusion protein [17] in order to identify potential downstream targets of BdbZIP10 during response to oxidative stress.

## Methods

### *Brachypodium* growing conditions and stress treatment

The production and characterization of bZIP10-GFP over-expressing *Brachypodium* was previously described [17]. Briefly, cDNA was prepared from oxidative-stressed leaf tissue of *Brachypodium distachyon* and was used as a template to amplify the bZIP10 gene (Bradi1g30140), which was inserted into a modified pART vector [22] under the control of the ZmUbi1 promoter, with a C-terminal GFP tag. The construct was transformed into *Agrobacterium* strain AGL1 [23], which was used to transform *Brachypodium distachyon*. Plants transformed with this ZmUbi1::bZIP10-GFP construct developed normally compared to the wild type plant [17].

Seeds of Bd21–3 or bZIP10-GFP transformed *Brachypodium distachyon* [17] were planted in SB40 Sunshine Growing Mix (Sun Gro Horticulture Inc., Bellevue, WA) with Osmocote Plus 15–9-12 (The Scotts Company, Marysville, OH) added at planting. After planting, the pots were placed at 4 °C with 8 h day length for 5 d to synchronize germination. Plants were then placed in a Conviron PGV36 growth chamber (Conviron, Winnipeg, Canada) set for 20 h light at 24 °C and 4 h dark at 18 °C. Plants were grown for at least six weeks post-germination before leaf tissue was harvested for ChIP sequencing and expression experiments. Paraquat treatments were performed using a modified version of existing leaf toxicity protocols [17, 24, 25]. For paraquat leaf treatment, 7- to 10-cm-long leaves were harvested, placed in fresh solutions of 160 μM paraquat, vacuum infiltrated for 5 min with house vacuum, and placed on a gentle rocker under continuous light for 24 h at room temperature. For ChIP, paraquat-treated leaf tissue was rinsed briefly in sterile distilled water prior to formaldehyde cross-linking with 1% formaldehyde in phosphate buffered saline (PBS) under vacuum infiltration for 20 min. Cross-linking was terminated by adding glycine (125 mM final concentration) directly to the samples and applying vacuum infiltration for an additional 10 min. The tissue was rinsed, blotted dry, and then ground to a fine powder in liquid nitrogen.

### ChIP-sequencing and ChIP-qPCR

ChIP-Seq service was performed by Zymo Research Corporation (Zymo Research Corp., Irvine, CA). Chromatin was prepared using the Zymo-Spin™ ChIP kit (Zymo Research Corp., Irvine, CA) following their modified protocol for isolation of plant chromatin. Sonication was performed at high power setting for 40 cycles (30 s on, 30 s off) using a Bioruptor Plus™ (Diagenode Inc., Denville, NJ), yielding a DNA fragment-size range of 200–700 bp. The ChIP assay was performed in triplicate (*N* = 3) using 12 μg of chromatin and 10 μg of anti-GFP ChIP Grade antibody (Abcam ab290, lot #GR240324–1; www.abcam.com) to target the bZIP10-GFP protein. Anti-IgG (Millipore, PP64B; Lot 2,565,474) at a concentration of 10 μg was included as a negative control. ChIP DNA was purified using the ChIP DNA Clean and Concentrator™ (Zymo Research Corp, Irvine, CA). ChIP-Seq libraries were prepared and sequenced on a HiSeq 1500 sequencer (Illumina, San Diego, CA). ChIP-Seq reads were aligned to the *Brachypodium diastachycon* reference genome (version 3.1) by Bowtie [26] with at most 2 mismatches. Reads that appeared more than twice at the same position on the same strand were discarded to remove PCR duplication. BigWig files were generated from the alignment for visualization purposes [27]. MACS2 (Model-based Analysis of ChIP-Seq) [28] was used to identify peaks using a q-value cutoff of 0.05.

In addition, ChIP-qPCR analysis was performed by Zymo Research Corporation. Briefly, 10 unique primer sets were designed to amplify a range of enriched regions identified in the GFP ChIP sequencing data. Anti-GFP ChIP and IgG negative control DNA enriched from two independent ChIP assays were amplified using ZymoTaq™ qPCR Premix (Zymo Research Corp., Irvine, CA). ChIP DNA enrichment was determined as % of input (i.e., the relative amount of immunoprecipitated DNA compared to 100% input DNA after qPCR analysis).

### Motif analysis

To identify possible binding motifs of the bZIP10 transcription factor, the ChIP peak sequences were subjected to MEME (Multiple EM for Motif Elicitation)-ChIP [29]. The MEME-ChIP program uses two ab initio motif discovery algorithms: MEME [30], and DREME (Discriminative Regular Expression Motif Elicitation) [31], which uses regular expressions to search for short eukaryotic TF motifs that are missed by MEME.

### Gene function of BdbZIP10 TF target genes

In order to determine putative functions of BdbZIP10 target genes, the set of 259 genes with ChIP-Seq peaks located in upstream promoter regions or potential downstream regulatory regions (2 kb upstream of transcription start site or 2 kb downstream of stop codon) or within annotated gene bodies were analyzed based on Gene Ontology (GO) categories using the AgriGO online Gene Set Enrichment Analysis (GSEA) tool [32, 33]. The default Fisher’s Exact Test and Benjamini-Yekutieli multiple test correction methods [34] were employed to generate *p*-values for statistical significance and corresponding False Discovery Rate (FDR) values.

Expression analysis of several genes with ChIP-fold enrichment greater than ten was examined to determine their expression levels in the presence or absence of oxidative stress (paraquat treatment). Three *BdbZIP10-GFP* OE lines (from three independent transformation events) were compared to three WT plants undergoing the same treatment (water or water with paraquat) for this analysis.

### RNA extraction and quantification

Three *BdbZIP10-GFP* OE lines (from independent transformation events) were compared to WT plants undergoing the same treatment for gene expression analysis. RNA extraction was performed with the Direct-zol™ RNA MiniPrep System (Zymo Research Corp., Irvine, CA). Briefly, 250 mg of leaf pieces were ground in liquid nitrogen, transferred to Trizol, and then column purified, including the DNase I digestion step, following the manufacturer’s instructions. Approximately 3 μg of RNA was used for cDNA synthesis using the SuperScript® III First-Strand Synthesis System (Invitrogen) following the manufacturer’s instructions.

Quantification of cDNA was performed using the BioRad Real Time PCR detection system and the Biorad iTaq™ Universal SYBR Green Supermix following the manufacturer’s instructions (Bio-Rad Laboratories, Hercules, CA). Primers were designed with QuantiPrime [35] using the website’s default settings, and tested for linearity. Primer sequences used in this study are listed in Additional file 1. For RT-qPCR, *BdUBC18* was used as a normalization housekeeping control gene [36] and the comparative C_T_ method [37] was used to analyze the data. The range of expression was calculated using the standard deviation of the ΔC_T_ value as described by Applied Biosystems in their “Guide to Performing Relative Quantitation of Gene expression Using Real-Time Quantitative PCR” [38].

## Results

### Analysis of ChIP-Seq peaks and validation with quantitative PCR

**Fig. 1:**
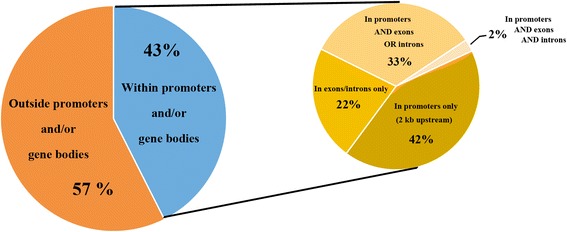
Distribution of ChIP peaks in the genome. Percent of peaks that reside 2 kb upstream of the transcriptional start site or 2 kb downstream of the stop codon (gene body), and location of peaks within the gene bodies

ChIP assays were performed using frozen cross-linked *Brachypodium* leaf tissue. The average fragment size of input and anti-GFP ChIP libraries were 371 and 400 bp, respectively. The input library had 14.4 million reads and the GFP Ab ChIP library had 5.6 million reads. Over 93% of the reads were mapped to the *Brachypodium* genome. The MACS2 (Model-based Analysis of ChIP-Seq) program [39] was used to identify enriched regions using a false discovery cutoff of 0.05. The location of the enriched peaks in the *Brachypodium* genome are presented in a supplemental table (Additional file 2). Of the 591 regions enriched, 43% of the peaks were located in genic regions (from 2 kb upstream of transcription start to 2 kb downstream of the stop codon including the coding region) (Fig. 1). Of peaks that were in genic regions, 42% were located only within promoter regions, 33% were located in promoter regions and in exons or introns, 22% were located in exons and introns only, and 2% were located in both promoter regions and introns and exons (Fig. 1). The genes associated with peaks in genic regions that were enriched greater than 10-fold with a known putative function are presented in Table 1.

**Table 1:** List of genes and their putative function

Gene Name	Putative Function	
Bradi1g06030	arachidonic acid binding (Blast2GO)	18.0
Bradi1g06882	PTHR31286:SF1 - NUCLEIC ACID BINDING / ZINC ION BINDING PROTEIN-RELATED	18.2
Bradi1g22650	leucine-rich repeat protein kinase	12.4
Bradi1g33347	PTHR24093:SF272 - CADMIUM/ZINC-TRANSPORTING ATPASE HMA1	11.4
Bradi1g35050	Glycosyl hydrolase (GH)	10.0
Bradi1g37100	PF01152 - Bacterial-like globin (Bac_globin)	25.4
Bradi1g37667	PTHR11040//PTHR11040:SF52 - ZINC/IRON TRANSPORTER // SUBFAMILY NOT NAMED	31.2
Bradi1g48360	F-Box	19.7
Bradi1g49020	PTHR14209//PTHR14209:SF7 - ISOAMYL ACETATE-HYDROLYZING ESTERASE 1	12.2
Bradi1g53680	high affinity zinc uptake transmembrane transporter activity (Blast2GO)	36.1
Bradi1g61170	glucosaminyl-phosphotidylinositol O-acyltransferase activity (Blast2GO)	12.5
Bradi1g68300	PTHR23310:SF14 - PROTEIN ACBP-7	13.0
Bradi1g74270	PF04720 - PDDEXK-like family of unknown function (PDDEXK_6)	15.7
Bradi2g07940	PF05558 - DREPP plasma membrane polypeptide (DREPP)	15.4
Bradi2g13590	endopeptidase activity (Blast2GO)	11.1
Bradi2g19620	PF04520 - Senescence regulator (Senescence_reg)	10.9
Bradi2g22520	zinc ion transmembrane transporter activity (Blast2GO)	28.6
Bradi2g22530	high affinity zinc uptake transmembrane transporter activity (Blast2GO)	31.4
Bradi2g23520	bHLH transcription factor	18.0
Bradi2g23560	PF00582 - Universal stress protein family (Usp)	11.3
Bradi2g25580	PTHR31251:SF2 - SQUAMOSA PROMOTER-BINDING-LIKE PROTEIN 7	24.1
Bradi2g25677	PTHR13268:SF0 - BREAST CARCINOMA-AMPLIFIED SEQUENCE 3	18.7
Bradi2g33110	zinc ion transmembrane transporter activity (Blast2GO)	32.3
Bradi2g44850	1.14.13.138 - Indolin-2-one monooxygenase / CYP71C2	19.1
Bradi2g50840	K14487 - auxin responsive GH3 gene family (GH3)	10.2
Bradi2g54670	GRAS transcription factor	13.7
Bradi2g59490	aspartate kinase activity (Blast2GO)	22.6
Bradi3g06780	KOG0156 - Cytochrome P450 CYP2 subfamily	10.6
Bradi3g11300	Putative glycosyltransferase belonging to CAZy family GT61	18.0
Bradi3g17900	zinc ion transmembrane transporter activity (Blast2GO)	39.2
Bradi3g28990	PTHR33083:SF9 - EMB	13.2
Bradi3g41600	PF07712 - Stress up-regulated Nod 19 (SURNod19)	10.6
Bradi3g47530	PF04570 - zinc-finger of the FCS-type	17.6
Bradi4g03530	RING, subfamily zinc finger (C3HC4-type RING finger) family protein	11.5
Bradi4g07210	1.1.99.35 - Soluble quinoprotein glucose dehydrogenase / Soluble glucose dehydrogenase	10.8
Bradi4g12190	phospholipid:diacylglycerol acyltransferase activity	11.1
Bradi4g25880	CAMK_KIN1/SNF1/Nim1_like.31 - CAMK includes calcium/calmodulin dependent protein kinases	12.0
Bradi4g42530	cation transmembrane transporter activity	14.1
Bradi4g44460	CAMK_KIN1/SNF1/Nim1_like.35 - CAMK includes calcium/calmodulin dependent protein kinases	12.3
Bradi5g18277	Prolyl oligopeptidase / Prolyl endopeptidase	30.3

The genes listed in this table are limited to those associated with peaks that were enriched greater than 10-fold and have been classified with a known function

**Fig. 2:**
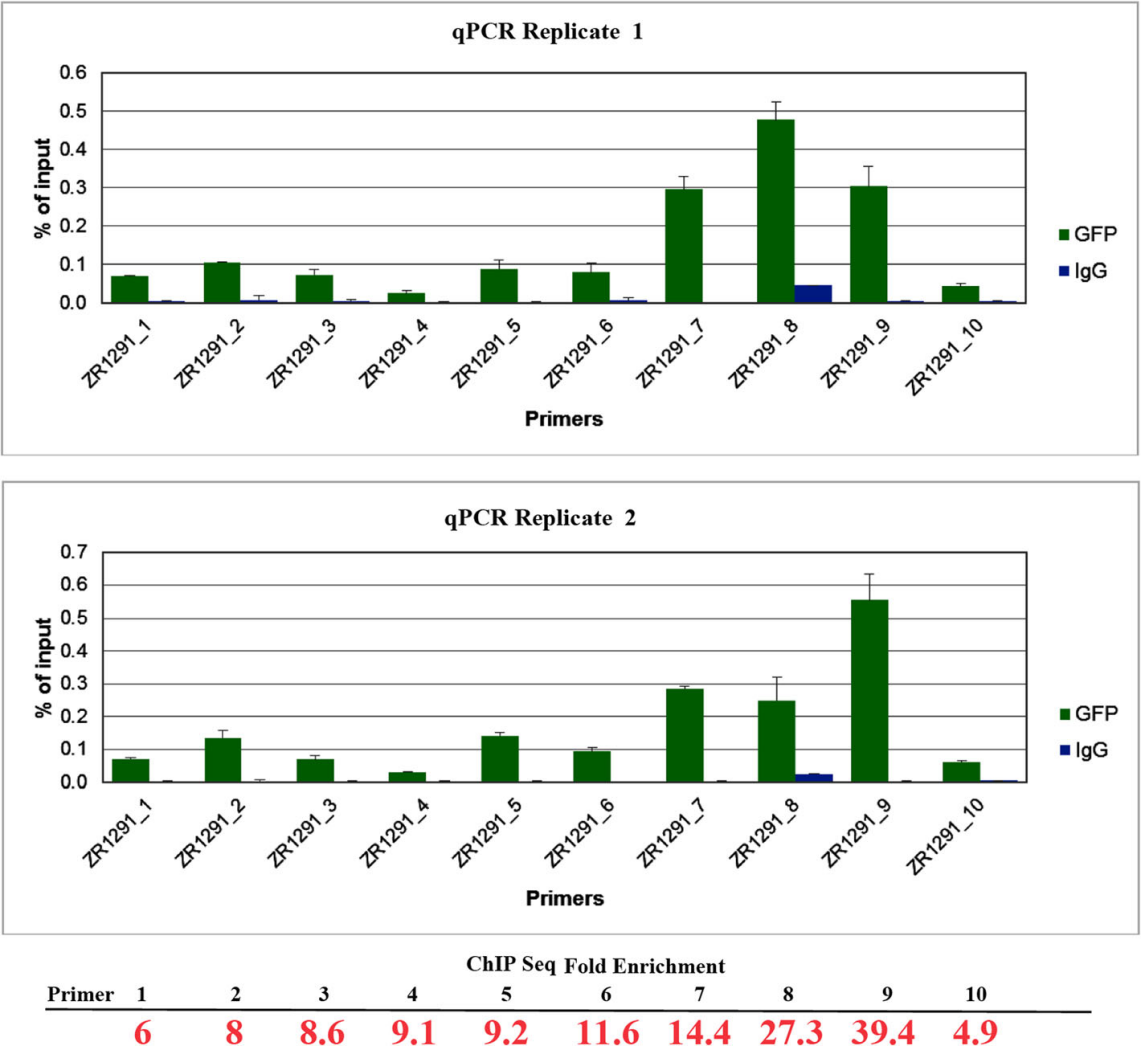
Verification of ChIP-Seq data with ChIP-qPCR. Two independent immunoprecipitates were analyzed with qPCR and compared to the ChIP-Seq data. The fold enrichment from the ChIP-Seq data is provided in red at the bottom of the image

To validate the ChIP-Seq results, ChIP-qPCR was performed on two independent paraquat-stressed leaf samples. Regions were chosen to target peaks with a range of fold-enrichments. The results are summarized in Fig. 2, with the ChIP-Seq fold enrichment of each called peak indicated below the graphs. While fold enrichment varied from 1.5–39.3, only peaks that were enriched at least 2.5-fold when compared to the input DNA were used for downstream analysis. The ChIP-qPCR values were in general agreement with the ChIP-Seq results.

### Motif analysis of bZIP10 target genes

**Fig. 3:**
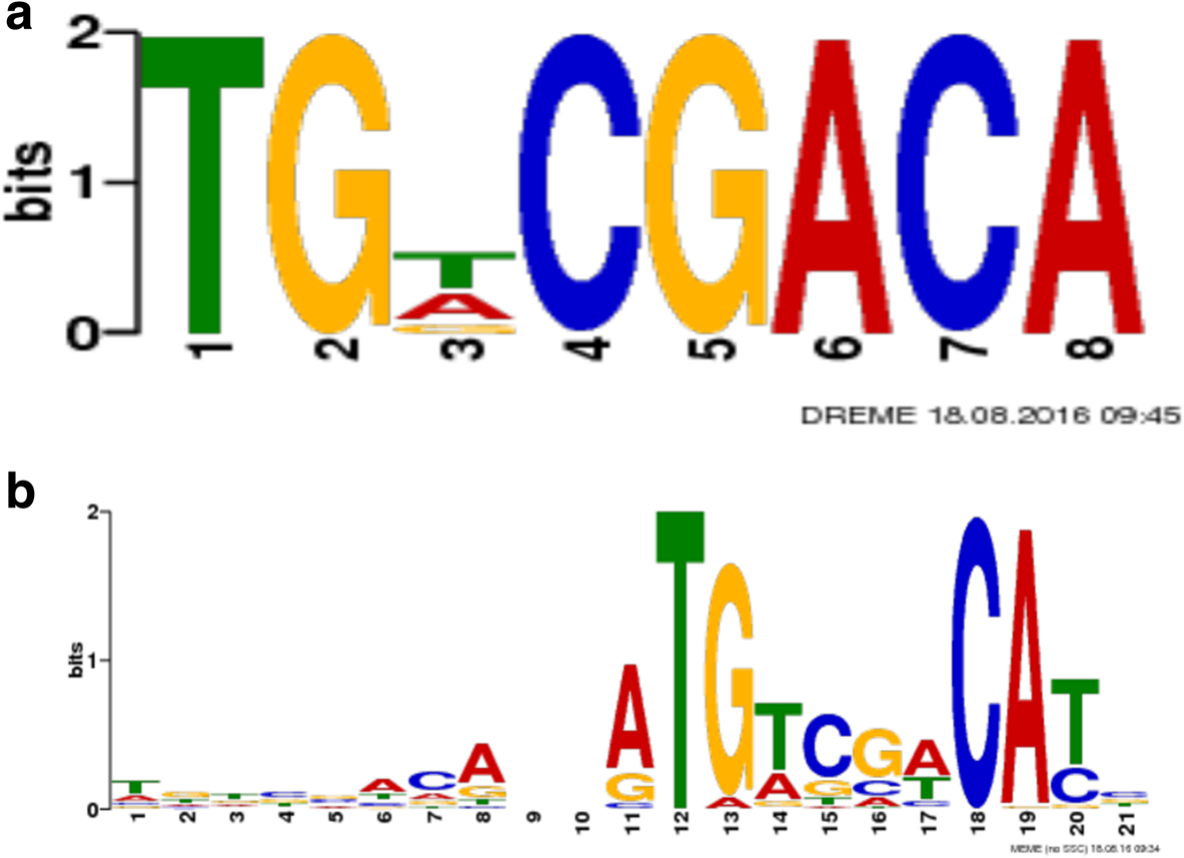
Consensus BdbZIP10-binding motif. The consensus sequences were determined by DREME (Discriminative Regular Expression Motif Elicitation; [2]) and MEME (Multiple Em for Motif Elicitation; [29]). Motifs identified with DREME (**a**) and MEME (**b**). The size of the letters in the figures is proportional to the frequency of the nucleotide in the motif

The most significantly enriched DREME [31] motif was TGDCGACA (*P*-value of 1.4e-33 and an E-value of 4.6e-29) (Fig. 3a), and this motif was distributed evenly among all chromosomes. The next most significant DREME motif (CGGYCGGA) was present mainly in the GC-rich region of chromosome 5 and may be an artifact. Three motifs were identified by MEME [29, 30]. Analysis of the location of the three MEME-identified motifs within the genome revealed that regions associated with MEME Motifs 2 and 3 were located in a ~ 206 Kb region on chromosome 5, between 2.3 Kb and 209 Kb. While these peaks were all below the 0.05 cutoff *p*-value, this region was notable for being GC-rich, as revealed with sequence motif searches. There is often a GC content bias in ChIP-Seq data [40–42]. At the same time, ChIP-Seq peaks have been shown to overlap GC-rich regions of the genome, and there is evidence that transcription factors tend to bind to GC-rich regions, even when they are not near transcription start sites [43]. Interestingly, this region of chromosome 5 contains small regions of homology to heat shock proteins as depicted in the PASA assembled ESTs in the genome browser [44], but there are very few genes that have been annotated in this region of chromosome 5. However, due to the high GC content in this region (bolded peaks in Additional file 2), these two MEME identified motifs are presumed to be false positives. MEME identified Motif 1 (Fig. 3b) was distributed evenly among all the chromosomes. The peaks identified by MEME and DREME are very similar, but with less discrimination in the central nucleotides in the binding site identified by MEME. Interestingly, none of the motifs found in this study contained the most reported bZIP binding ACGT core sequence, which is present in different bZIP binding sites, including the G-box (CACGTG); C-box (GACGTC); and A-box (TACGTA) motifs reported for bZIP proteins [8, 45]. However, the DREME motif, TGDCGACA, does share homology to the *Arabidopsis* zinc deficiency response element (RTGTCGACAY) previously reported [13]. The ChIP enriched sequences were examined for the presence of the identified MEME motif or for motifs related to binding sites for other bZIP transcription factors, such as the C, A or G boxes [8, 45]. Several of the peaks enriched greater than 10-fold did not contain the identified motif, but some did contain ACGT core motifs, suggesting the possibility of heterodimerization between the bZIP10 TF and other bZIP TFs [46].

### Gene ontology analysis to identify biological and functional enriched categories

**Fig. 4:**
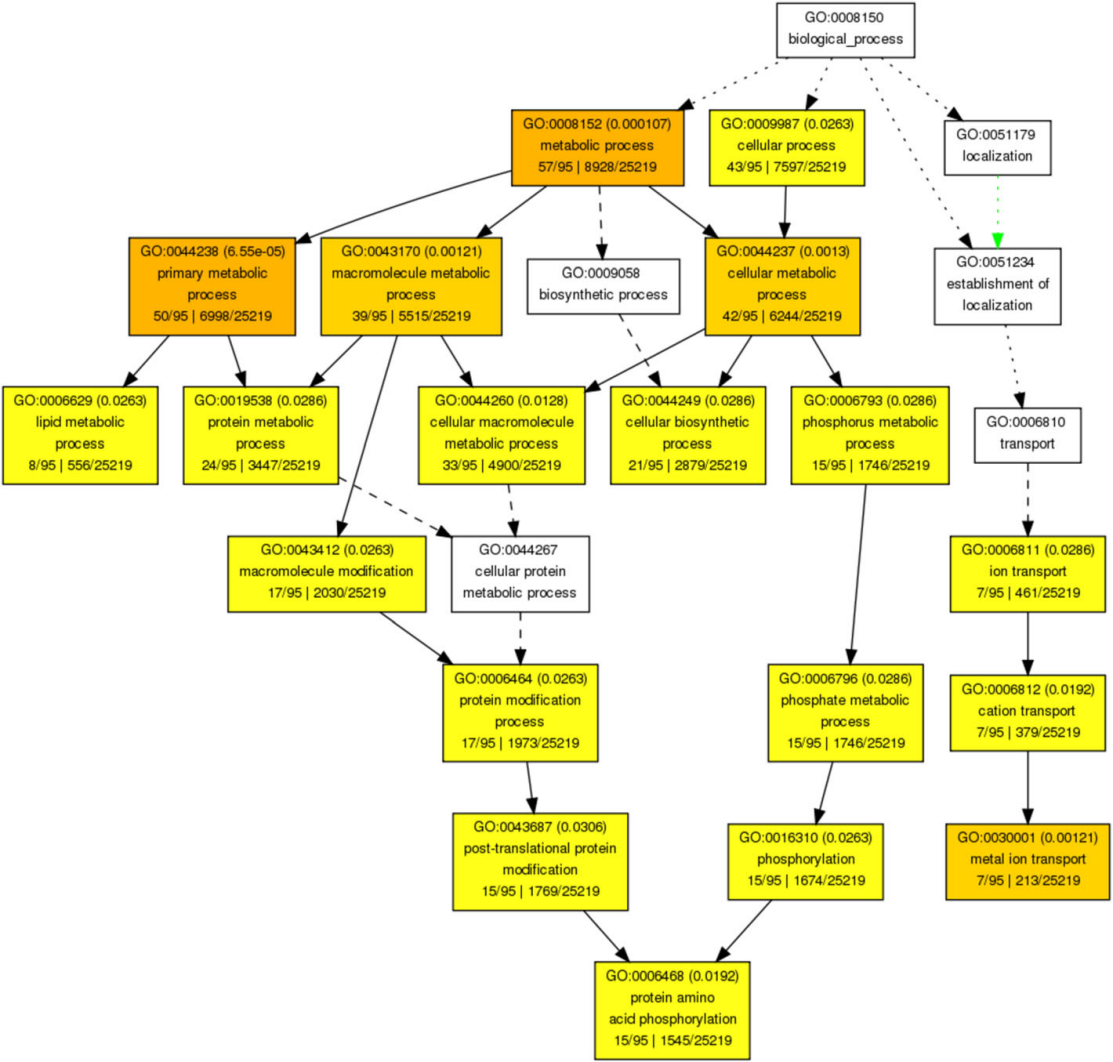
Functional categories of BdbZIP10-bound genes. Each square represent a single gene ontology class. White boxes were not significantly enriched. Yellow boxes represent groups of genes that are significantly enriched at 0.01 < *P* < 0.05. Orange boxes are significant at *P* < 0.01

Gene Ontology (GO) studies using the AgriGO online Gene Set Enrichment Analysis (GSEA) tool (http://bioinfo.cau.edu.cn/agriGO/analysis.php) [32, 33] revealed 19 GO categories belonging to the Biological Process (BP) ontology that were determined to be significantly over-represented in the ChIP-Seq sample relative to the *Brachypodium* genome (Additional file 3, Fig. 4). Gene ontology categories significantly enriched (*p*-value < 0.01; orange colored boxes in Fig. 4) for biological processes included metabolic processes (cellular, primary and macromolecular) and metal ion transport. Additional categories were identified when using a *P* value greater than 0.01 and less than 0.05 (Fig. 4; yellow boxes). Four biological function categories were significantly enriched with (False Discovery Rates) FDRs and *p*-values < 0.01: metal ion transmembrane transporter, transferase, catalytic, and binding activities (Additional file 3). Several other functional categories including kinases and phosphotransferases, cation/ion transmembrane transporters, transferases (phosphorus-containing and glycosyl groups), and some nucleoside/nucleotide binding activities were also enriched with *P* values between 0.01 and 0.05. Members of each enriched GO category are listed in Additional file 3.

### Expression analysis of genes from enriched Gene Ontology (GO) categories

Expression levels of several genes from the enriched GO categories with ChIP-fold enrichment greater than ten were analyzed in the presence (paraquat treated) or absence (water treated) of oxidative stress in *BdbZIP10-GFP* OE and WT plants (Table 2). Since a value of one represents equivalent expression, ranges that surrounded, but included one, were not considered to have significantly different expression. Five genes related to zinc transport were analyzed and four of these genes were found to be up-regulated in nonstressed *BdZIP10* OE compared to nonstressed WT plants (Bradi2g33110.1, Bradi2g22520.1, Bradi1g53680.1, Bradi3g17900.1), while expression of Bradi5g21580.1 was similar in nonstressed WT and *BdbZIP10* OE plants (Table 2). These zinc transporter genes were down regulated in *BdbZIP10-GFP* OE plants treated with paraquat compared to the transgenic control treatment, but were variable in their response in WT plants treated with paraquat compared to control WT plants; Bradi2g33110.1 was down regulated, Bradi1g53680.1 tended towards down regulation, and Bradi2g22520.1 was upregulated, while the other genes had equivalent expression. All of these genes, except Bradi5g21580.1, were increased in *BdbZIP10-GFP* OE plants when compared to WT under both control and oxidative stress conditions. Expression of Bradi2g22520.1 and Bradi1g53680.1, both having zinc ion transmembrane transporter activity, were increased over 30 fold in *BdbZIP10-GFP* OE plants compared to WT under control conditions. Some genes, such as Bradi1g68300 (Acyl-CoA-Binding protein) were similarly induced in both the *BdbZIP10-GFP* OE and WT under oxidative stress (paraquat treatment), but showed no expression differences between transgenic and WT plants under stressed and control conditions (Table 2). Similarly, a stress up-regulated Nod19 gene was increased in both *BdbZIP10-GFP* OE and WT plants when exposed to paraquat, with slightly higher induction in the WT plants; and slightly increased expression in *BdbZIP10-GFP* OE plants compared to WT plants under control conditions (Table 2). A bacterial-like globin gene (Bradi1g37100) showed slightly reduced expression in the transgenic plants when treated with paraquat compared to transgenics treated with water, and was expressed at higher levels in the *BdbZIP10-GFP* OE plants compared to the WT under both control and paraquat conditions (Table 2). Expression of an isoamyl acetate-hydrolyzing esterase (Bradi1g49020) was increased in WT plants and slightly decreased in *BdbZIP10-GFP* OE plants treated with paraquat compared to untreated plants. Transgenic *BdbZIP10-GFP* OE plants had increased isoamyl acetate-hydrolyzing esterase expression levels compared to WT plants in the control treatment and slightly decreased levels with paraquat treatment (Table 2).

**Table 2:** Expression analysis of select genes

	Paraquat treatment compared to water		*BdbZIP10-GFP* OE Compared to WT		Putative function Phytozome/Panther
Gene Name	*BdbZIP10*-*GFP* OE	WT	Water	Paraquat	
Bradi2g33110.1	0.2–0.48	0.24–0.33	**2.0–4.1**	**2.1–4.9**	**Bradi2g33110.1.p - zinc ion transmembrane transporter activity**
Bradi2g22520.1	0.28–0.62	2.1–2.8	**52.8–93.1**	**8.1–18.2**	**Zinc ion transmembrane transporter activity**
Bradi1g53680.1	0.05–0.14	0.24–1.17	**30–123**	**5.4–18.1**	**High affinity zinc uptake** **transmembrane transporter activity**
Bradi3g17900.1	0.27–0.68	0.6–1.2	**6.4–14.8**	**3.1–7.8**	**Zinc ion transmembrane** **transporter activity**
Bradi5g21580.1	0.16–0.25	0.8–1.2	1.1–2.4	1.0–1.4	High affinity zinc uptake transmembrane transporter activity
Bradi1g68300	3.9–5.2	3.9–5.1	0.8–1.2	0.9–1.3	PTHR23310:SF14-Protein ACBP-7
Bradi1g37100	0.59–0.81	0.72–1.63	**2.7–5.9**	**2.1–3.0**	**PF01152 – Bacterial-like globin; Thoredoxin peroxidase**
Bradi1g49020	0.4–0.6	3.3–7.4	**3.4–8.0**	**0.4–0.6**	**PTHR14209//PTHR14209:SF7- Isoamyl acetate-hydrolyzing esterase 1**
Bradi3g41600	3.9–5.2	7.8–13.1	1.2–1.7	0.5–0.7	Stress up-regulated Nod19 (SURNod19)

Comparison of gene expression levels between paraquat and water treated *BdbZIP10* OE and wildtype plants. Gene expression level of *BdbZIP10-GFP* OE compared to wildtype plants. Numbers indicate the range of three independently *BdbZIP10-GFP* OE transformed lines, calculated using the standard deviation of the C_t_ values. Bold text indicates the most significant changes between *BdbZIP10-GFP* OE and wildtype plants

## Discussion

Zinc is an essential micronutrient in many cellular processes, as a cofactor for many enzymes and transcription factors, as a structural component of proteins, and as a signaling molecule. It is also thought to be involved in protecting the cell from oxidative stress [47, 48]. It is therefore essential that organisms have the capacity to maintain zinc homeostasis within the cell and within the organism through zinc uptake, transport, storage, exclusion and/or compartmentalization [49].

**Fig. 5:**
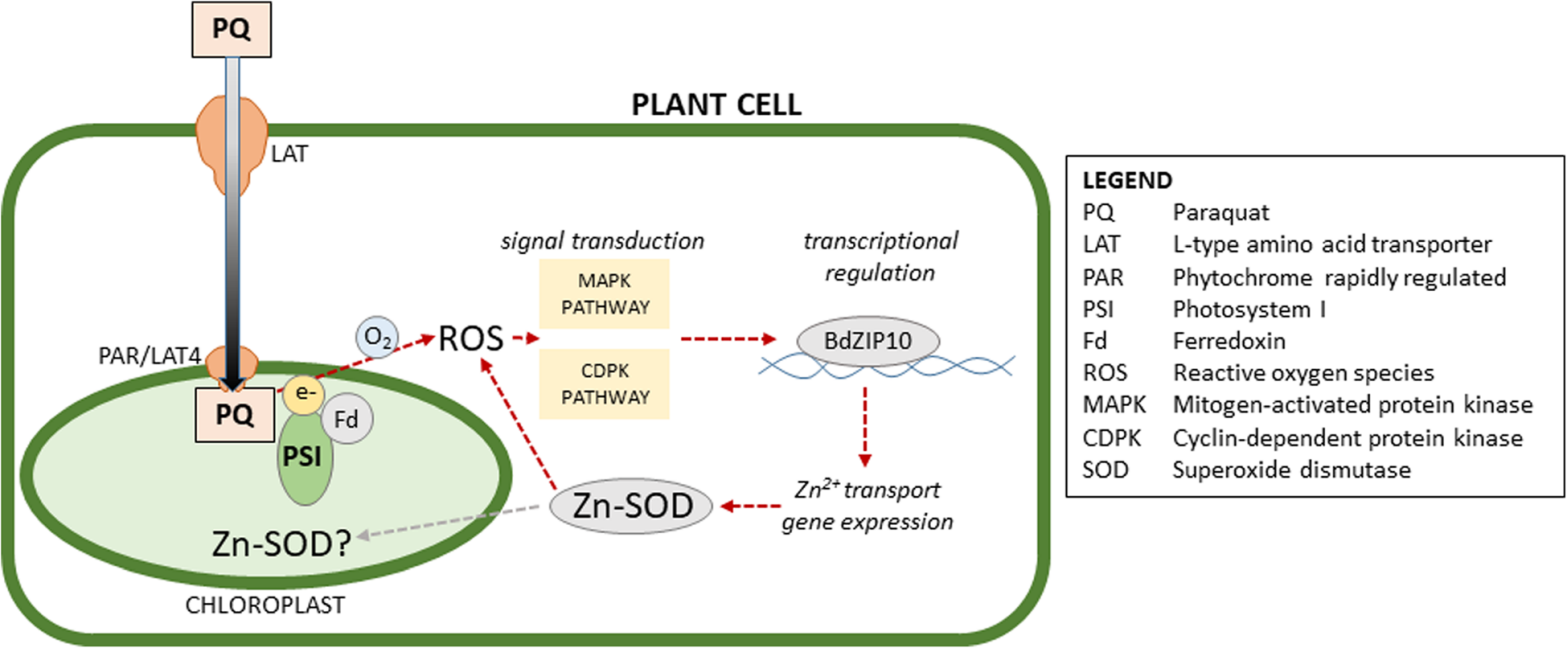
Proposed role of BdbZIP10 in response to paraquat-induced oxidative stress. Paraquat entry into plant chloroplasts results in production of reactive oxygen species, which in turn initiate protein kinase signaling pathways. Phosphorylation cascades activate transcription factors such as BdbZIP10. Overexpression of BdbZIP10 further upregulates genes controlling intracellular zinc transport, increasing activity of cytosolic and/or chloroplastic superoxide dismutases that have Zn^2+^ as a cofactor

Paraquat enters plant cells, then chloroplasts, via plasma membrane-bound homologs of polyamine transporters, where it strongly induces oxidative stress by production of reactive oxygen species (Fig. 5) [50]. Accumulation of ROS initiates an abiotic stress response, including such phosphorylation-based signal transduction pathways as mitogen-acitvated protein kinases (MAPKs) and cyclin-dependent protein kinases (CDPKs) [51]. Signal transduction leads to the activation of transcription factors that upregulate expression of key stress response genes.

Superoxide dismutases (SODs) constitute an important part of the plant cell’s defense against ROS. SODs are categorized into three groups according to their metal cofactors (iron, manganese, copper-zinc), and those in the copper-zinc category are found throughout the plant cell [52]. Therefore, the abiotic stress response and zinc homeostasis are closely connected.

In *Arabidopsis*, two transcription factors, AtbZIP19 and AtbZIP23, have been reported to regulate the zinc deficiency response [13], but a later report indicated that the single *Atbzip19* mutant was hypersensitive to zinc deficiency, and they concluded that AtbZIP19 is involved in response to Zn deficiency, while AtbZIP23 is important for maintaining Zn homeostasis [14]. They found two Zn transporters and three defensin-like family proteins that were up-regulated in WT compared to *Atbzip19* mutants on zinc deficient media, and they suggested that these proteins are responsible for the hypersensitivity of the *Atbzip19* mutant to zinc deficiency [14].

An excess of zinc is also detrimental to plants, but there are species such as *Arabidopsis halleri* (L.) O’Kane and Al-Shehbaz ssp. *halleri* that are Zn hyperaccumulators and are able to grow in metal-contaminated soils [53, 54]. Studies comparing *A. halleri* to *A. thaliana* revealed high constitutive expression of genes involved in metal homeostasis. These included genes coding for ZIP transporter proteins and proteins that may function in cellular Zn sequestration and detoxification such as putative P-type metal ATPase, cation diffusion facilitator, and nicotianamine synthase genes [55, 56]. Plant defensins were also reported to play a role in tolerance to excess Zn in the Zn hyper-accumulator *Arabidopsis halleri* and it was suggested they may play a role in metal physiology [57]. These genes are likely essential for maintaining zinc homeostasis in plants.

Overexpression of a *Brachypodium* homolog of *AtbZIP19/23*, *BdbZIP10*, enhanced oxidative stress resistance in *Brachypodium*, likely through the activation of detoxification and protective oxidative stress genes [17]. We performed ChIP sequence analysis to identify binding motifs and potential target genes of BdbZIP10 when plants were exposed to paraquat-induced oxidative stress. A binding motif similar to the zinc deficiency response element (ZDRE) was identified (Fig. 3), which is not unexpected, as *Brachypodium BdbZIP10* is most homologous to *AtbZIP 19* and *23*, which have been shown to bind to the ZDRE. Many of the target genes identified are involved in zinc homeostasis. Five genes with zinc transporter activity were shown to be highly enriched in the ChIP sequences, but not all of these showed differential expression between WT and *BdbZIP10-GFP* OE plants (Tables 1 and 2). Data available for these zinc transporter genes in Phytozome [44] revealed that most were also found to be differentially expressed during some other type of stress - cold, salt, drought, or heat stress; except one of the zinc ion transporters (Bradi2g33110.1), which was differentially expressed in root tissues exposed to different nitrogen compounds [44]. It is possible that the zinc transporter genes that were not upregulated in response to paraquat-induced oxidative stress could require additional TFs for induction, may be localized in the roots, or could be induced in response to zinc deficiency, but not oxidative stress. It will be interesting to look at the effect of high and low levels of zinc in *BdbZIP10* OE plants to determine if a subset of zinc transporter genes might be differentially regulated during zinc stress, as compared those identified during oxidative stress. A Cd/Zn-transporting ATPase HMA1 was also present in the ChIP-Seq enriched peaks (Table 1). This protein is localized in the chloroplast and is thought to control Zn content in plastids, is involved in Cd detoxification, and functions as a Ca/heavy metal pump [58]. Furthermore, *Arabidopsis HMA1* knockout plants were more sensitive to Zn than WT plants [59], suggesting the importance of this enzyme in Zn homeostasis. An Acyl CoA binding protein was also among the ChIP-enriched sequences. Besides their role in phospholipid metabolism and signaling, it has been suggested that members of this class of proteins may also play a role in heavy metal binding [60]. The fact that this protein was induced in both WT and *BdbZIP10-GFP* OE plants when exposed to paraquat may indicate that it plays a role in signaling during oxidative stress.

There were several different classes of compounds involved in oxido-reductase reactions that were enriched in the ChIP-Seq genes. A bacterial-like globin gene/thioredoxin peroxidase, which interacts with oxygen was also identified (Table 1) and found to be upregulated in the Bd*bZIP10-GFP* OE compared to WT plants under both stressed and control conditions (Table 2). A stress up-regulated Nod 19 gene that was previously shown to be important in beans to cope with abiotic and biotic generated oxidative stress [61] was enriched in the ChIP-Seq peaks. This gene was found to be upregulated in both the WT and Bd*bdbZIP10-GFP* OE plants when exposed to paraquat treatment, but there was little difference between the transgenic lines and the WT plants under both control and paraquat conditions. Cytochrome P450s, which are involved in assorted biosynthetic pathways and in detoxification of multiple classes of compounds including heavy metals and herbicides [62], were also identified in our ChIP-Seq library, however, the identified MEME motif was not present in the promoter of this gene. Several other genes also lacked the ZDRE-like motif and did not contain any of the other bZIP binding motifs. It is possible that BdbZIP10 interacts with other TFs/signals present during oxidative stress not related to this motif, or alternatively this could be an artifact.

There were also some genes that were ChIP-Seq enriched that are not directly involved in zinc transport, but are related to lipid metabolism and gene regulation. A putative SF7-Isoamyl acetate-hydrolyzing esterase (Pfam: GDSL-like Lipase/Acylhydrolase) was upregulated in the absence of paraquat in the Bd*bZIP10-GFP* OE plants compared to WT plants, and was also induced in WT when exposed to paraquat (Table 2). Many GDSL esterase/lipases are involved in biotic and abiotic stress responses, and members of this family are also involved in plant growth and development and defense responses [63]. This gene did not contain the identified MEME binding site, but it did contain an ACGT motif binding site. Another gene coding for an enzyme involved in triacylglycerol (TAG) synthesis was present in the ChIP-Seq enriched genes (Bradi4g12190, phospholipid:diacylglycerol acyltransferase), but there were no bZIP binding sites in the ChIPed peak sequence, suggesting this may be an artifact, or that there are alternative binding sites/interactions at play. Triacylglycerol synthesis is involved in maintaining membrane lipid homeostasis [64] and protecting cells from oxidative damage during lipid peroxidation [65, 66]. It would be an interesting feedback mechanism if a zinc deficiency TF helps to regulate genes that are involved in membrane stability, as zinc deficiency leads to oxidative stress and eventually membrane destabilization.

## Conclusion

The BdbZIP10 (Bradi1g30140) protein from *Brachypodium* is most closely related to the Group 1 members of the F family of bZIP transcription factors represented by AtbZIP19 and 23. Members of this group of TFs, the ZIP transporters to which they bind, and the zinc deficiency responsive element (ZDRE) are highly conserved across land plants, suggesting they play a vital role in maintaining zinc homeostasis in plants [12, 13]. Members of F subgroup 2, which includes AtbZIP24, are more variable across land plants, with monocots generally having more members, but with some plant species lacking members in this subgroup [12]. The conservation across species of bZIP F group 1 members and their relatively higher levels of expression suggest they may serve an essential role in land plants. Members of the bZIP F group 1 are involved in maintaining zinc homeostasis in plants. Zinc deficiency causes decreased crop yields, reduced crop nutritional value, and also contributes to major health problems in humans [47, 48]. Zinc is an important cofactor for many enzymes and transcription factors, including SODs that detoxify ROS during the abiotic stress response (52). Zinc deficiency results in oxidative stress in animal and plant systems [48, 49, 67]. In this study, we identified potential targets of the BdbZIP10 TF in response to paraquat-induced oxidative stress and found a motif that is different from most reported bZIP TF motifs, but with strong homology to the *Arabidopsis* ZDRE. This, coupled with the fact that our ChIP analysis identified a number of zinc transporter genes suggests that BdbZIP10 is potentially involved in zinc homeostasis, as it relates to oxidative stress. Overexpression of BdbZIP10 may boost cellular Cu-Zn-SOD activity, increasing protection against ROS production. TF motifs play a key role in transcriptional regulation. This study contributes to a better understanding of the role BdbZIP10 plays in oxidative stress in *Brachypodium*. To truly understand an organism’s response to any particular signal or stress, it is crucial to identify the control elements involved in the signaling and response pathways. In addition to the zinc transporter genes, other genes associated with signal transduction pathways, such as phosphatases and kinases were also identified in our ChIP analysis. It will be interesting to investigate the role that these proteins play in the signaling pathways associated with or regulated by BdbZIP10. Our future research will focus on determining the role BdbZIP10 plays in other abiotic stresses.

## Additional files

Additional file 1: Primers used in this study. Primers used for gene expression analysis and their calculated efficiency. Zymo Primers used for ChIP-qPCR analysis. (DOCX 41 kb)

Additional file 2: List of enriched peaks and their location in the *Brachypodium* genome. (XLSX 65 kb)

Additional file 3: Gene ontology categories and their gene members represented in ChIP sequences. Gene ontology (GO) groups, based on biological processes and molecular functions, with False Discovery Rates (FDR) < 0.01 are bolded, FDR > 0.01 and < 0.05 are italicized, and FDR > 0.05 and < 1 and *P* values < 0.05 are in normal font. (DOCX 27 kb)

## Abbreviations

At: *Arabidopsis thaliana*
Bd: *Brachypodium*
ChIP: chromatin immunoprecipitation
C_T_: cycle threshold
DREME: Discriminative Regular Expression Motif Elicitation
GFP: Green fluorescent protein
GO: Gene Ontology
MEME: Multiple EM for Motif Elicitation
RT: reverse transcriptase
Seq: sequencing
TF: transcription factor
