# Supplementary material for: Genome-wide (ChIP-seq) identification of target genes regulated by BdbZIP10 during paraquat-induced oxidative stress
Source: BMC Plant Biol. 2018 Apr 10;18:58. doi: 10.1186/s12870-018-1275-8 (PMC5894230; doi:10.1186/s12870-018-1275-8)
Supplement: Supplementary file 1 — Primers used in this study. Primers used for gene expression analysis and their calculated efficiency. Zymo Primers used for ChIP-qPCR analysis. (DOCX 41 kb) [file 12870_2018_1275_MOESM1_ESM.docx]

**Additional File 1.** Primers used in this study. Primers used for gene expression analysis and their calculated efficiency. Zymo Primers used for ChIP-qPCR analysis.
