# Supplementary material for: Genome-wide (ChIP-seq) identification of target genes regulated by BdbZIP10 during paraquat-induced oxidative stress
Source: BMC Plant Biol. 2018 Apr 10;18:58. doi: 10.1186/s12870-018-1275-8 (PMC5894230; doi:10.1186/s12870-018-1275-8)
Supplement: Supplementary file 3 — Gene ontology categories and their gene members represented in ChIP sequences. Gene ontology (GO) groups, based on biological processes and molecular functions, with False Discovery Rates (FDR) < 0.01 are bolded, FDR > 0.01 and < 0.05 are italicized, and FDR > 0.05 and < 1 and P values < 0.05 are in normal font. (DOCX 27 kb) [file 12870_2018_1275_MOESM3_ESM.docx]

**Additional file 3.** List of genes in each of the enriched gene ontology (GO) categories. Bolded categories are significant at p<0.01 for both the p-value and false discovery rate (FDR). Categories that are significant with p-values <0.01 and FDR between 0.01 and 0.05 are in italics. The categories in normal font have p-values <0.05.

**GO:Accession Term Type Term**

**Query Query Bd gen Bd gen P-value FDR**

**Biological Processes Items Total Items Total Entries**

**GO:0044238 P primary metabolic process**

**50 95 6998 25219 2.9e-07 6.5e-05**

**Bradi2g51560.1 // Bradi2g52330.1 // Bradi1g48880.1 // Bradi2g36470.1 // Bradi4g25880.1 // Bradi1g18490.1 // Bradi3g57100.1 // Bradi2g48830.1 // Bradi1g45160.1 // Bradi4g44460.1 // Bradi1g48870.1 // Bradi2g46560.1 // Bradi4g45010.1 // Bradi2g50140.1 // Bradi4g35910.1 // Bradi1g21420.1 // Bradi4g39480.1 // Bradi3g44640.1 // Bradi3g56070.1 // Bradi2g49420.1 // Bradi4g19060.1 // Bradi1g17270.1 // Bradi5g16590.1 // Bradi2g24090.1 // Bradi4g07930.1 // Bradi4g35780.1 // Bradi2g02510.1 // Bradi1g63600.1 // Bradi1g22650.1 // Bradi5g23680.1 // Bradi2g52380.1 // Bradi2g59490.1 // Bradi2g47220.1 // Bradi4g13280.1 // Bradi3g21550.1 // Bradi3g37600.1 // Bradi1g49020.1 // Bradi1g73720.1 // Bradi3g48880.1 // Bradi2g23520.1 // Bradi1g61170.1 // Bradi4g44760.1 // Bradi4g29600.1 // Bradi1g51540.1 // Bradi3g18580.1 // Bradi5g23890.1 // Bradi3g08660.1 // Bradi3g48230.1 // Bradi4g21980.1 // Bradi1g60170.1**

**GO:0008152 P metabolic process**

**57 95 8928 25219 9.6e-07 0.00011**

**Bradi2g51560.1 // Bradi2g52330.1 // Bradi1g48880.1 // Bradi2g36470.1 // Bradi4g25880.1 // Bradi1g18490.1 // Bradi3g57100.1 // Bradi2g48830.1 // Bradi1g45160.1 // Bradi4g44460.1 // Bradi1g48870.1 // Bradi1g72180.1 // Bradi2g46560.1 // Bradi4g45010.1 // Bradi2g50140.1 // Bradi4g35910.1 // Bradi1g21420.1 // Bradi4g39480.1 // Bradi3g44640.1 // Bradi3g56070.1 // Bradi2g49420.1 // Bradi4g19060.1 // Bradi1g17270.1 // Bradi5g23680.1 // Bradi2g16290.1 // Bradi5g16440.1 // Bradi2g24090.1 // Bradi4g07930.1 // Bradi5g16590.1 // Bradi4g35780.1 // Bradi5g22830.1 // Bradi2g02510.1 // Bradi5g16120.1 // Bradi1g63600.1 // Bradi1g22650.1 // Bradi5g04070.1 // Bradi2g52380.1 // Bradi2g59490.1 // Bradi2g47220.1 // Bradi4g13280.1 // Bradi3g21550.1 // Bradi3g37600.1 // Bradi1g49020.1 // Bradi4g12190.1 // Bradi1g73720.1 // Bradi3g48880.1 // Bradi2g23520.1 // Bradi1g61170.1 // Bradi4g44760.1 // Bradi4g29600.1 // Bradi1g51540.1 // Bradi3g18580.1 // Bradi5g23890.1 // Bradi3g08660.1 // Bradi3g48230.1 // Bradi4g21980.1 // Bradi1g60170.1**

**GO:0030001 P metal ion transport**

**7 95 213 25219 2e-05 0.0012**

**Bradi1g74680.1 // Bradi2g33110.1 // Bradi3g17900.1 // Bradi5g21580.1 // Bradi1g53680.1 // Bradi2g22530.1 // Bradi2g22520.1**

**GO:0043170 P macromolecule metabolic process**

**39 95 5515 25219 2.1e-05 0.0012**

**Bradi2g51560.1 // Bradi2g52330.1 // Bradi1g48880.1 // Bradi2g36470.1 // Bradi4g25880.1 // Bradi1g60170.1 // Bradi2g48830.1 // Bradi1g45160.1 // Bradi4g44460.1 // Bradi2g46560.1 // Bradi2g50140.1 // Bradi4g35910.1 // Bradi4g39480.1 // Bradi3g44640.1 // Bradi2g49420.1 // Bradi4g19060.1 // Bradi1g17270.1 // Bradi5g16590.1 // Bradi4g07930.1 // Bradi4g35780.1 // Bradi2g02510.1 // Bradi1g63600.1 // Bradi1g22650.1 // Bradi2g52380.1 // Bradi2g47220.1 // Bradi4g13280.1 // Bradi3g21550.1 // Bradi3g37600.1 // Bradi1g73720.1 // Bradi3g48880.1 // Bradi2g23520.1 // Bradi1g61170.1 // Bradi4g44760.1 // Bradi1g51540.1 // Bradi3g18580.1 // Bradi5g23890.1 // Bradi3g08660.1 // Bradi3g48230.1 // Bradi4g21980.1**

**GO:0044237 P cellular metabolic process**

**42 95 6244 25219 2.9e-05 0.0013**

**Bradi2g51560.1 // Bradi2g52330.1 // Bradi1g48880.1 // Bradi2g36470.1 // Bradi4g25880.1 // Bradi1g60170.1 // Bradi2g48830.1 // Bradi4g44460.1 // Bradi1g72180.1 // Bradi4g45010.1 // Bradi2g50140.1 // Bradi4g35910.1 // Bradi1g21420.1 // Bradi4g39480.1 // Bradi3g56070.1 // Bradi2g49420.1 // Bradi4g19060.1 // Bradi1g17270.1 // Bradi2g16290.1 // Bradi5g16440.1 // Bradi4g07930.1 // Bradi5g16590.1 // Bradi4g35780.1 // Bradi5g22830.1 // Bradi2g02510.1 // Bradi1g63600.1 // Bradi1g22650.1 // Bradi5g23680.1 // Bradi2g52380.1 // Bradi2g59490.1 // Bradi2g47220.1 // Bradi3g37600.1 // Bradi1g73720.1 // Bradi3g48880.1 // Bradi2g23520.1 // Bradi1g61170.1 // Bradi4g44760.1 // Bradi1g51540.1 // Bradi3g18580.1 // Bradi5g23890.1 // Bradi3g08660.1 // Bradi4g21980.1**

*GO:0044260 P cellular macromolecule metabolic process*

*33 95 4900 25219 0.00034 0.013*

*Bradi2g51560.1 // Bradi2g52330.1 // Bradi1g48880.1 // Bradi2g36470.1 // Bradi4g25880.1 // Bradi3g37600.1 // Bradi2g48830.1 // Bradi4g44460.1 // Bradi2g50140.1 // Bradi4g35910.1 // Bradi4g39480.1 // Bradi2g49420.1 // Bradi4g19060.1 // Bradi1g17270.1 // Bradi5g16590.1 // Bradi4g07930.1 // Bradi4g35780.1 // Bradi2g02510.1 // Bradi1g63600.1 // Bradi1g22650.1 // Bradi2g52380.1 // Bradi2g47220.1 // Bradi1g60170.1 // Bradi1g73720.1 // Bradi3g48880.1 // Bradi2g23520.1 // Bradi1g61170.1 // Bradi4g44760.1 // Bradi1g51540.1 // Bradi3g18580.1 // Bradi5g23890.1 // Bradi3g08660.1 // Bradi4g21980.1*

*GO:0006468 P protein amino acid phosphorylation*

*15 95 1545 25219 0.00068 0.019*

*Bradi1g22650.1 // Bradi2g36470.1 // Bradi4g25880.1 // Bradi2g51560.1 // Bradi1g60170.1 // Bradi2g48830.1 // Bradi4g07930.1 // Bradi5g23890.1 // Bradi4g44460.1 // Bradi3g08660.1 // Bradi5g16590.1 // Bradi3g37600.1 // Bradi4g21980.1 // Bradi1g17270.1 // Bradi2g02510.1*

*GO:0006812 P cation transport*

*7 95 379 25219 0.00063 0.019*

*Bradi1g74680.1 // Bradi2g33110.1 // Bradi3g17900.1 // Bradi5g21580.1 // Bradi1g53680.1 // Bradi2g22530.1 // Bradi2g22520.1*

*GO:0016310 P phosphorylation*

*15 95 1674 25219 0.0015 0.026*

*Bradi1g22650.1 // Bradi2g36470.1 // Bradi4g25880.1 // Bradi2g51560.1 // Bradi1g60170.1 // Bradi2g48830.1 // Bradi4g07930.1 // Bradi5g23890.1 // Bradi4g44460.1 // Bradi3g08660.1 // Bradi5g16590.1 // Bradi3g37600.1 // Bradi4g21980.1 // Bradi1g17270.1 // Bradi2g02510.1*

*GO:0009987 P cellular process*

*43 95 7597 25219 0.0013 0.026*

*Bradi2g51560.1 // Bradi2g52330.1 // Bradi1g48880.1 // Bradi2g36470.1 // Bradi4g25880.1 // Bradi1g60170.1 // Bradi2g48830.1 // Bradi4g44460.1 // Bradi1g72180.1 // Bradi4g45010.1 // Bradi2g50140.1 // Bradi4g35910.1 // Bradi1g21420.1 // Bradi4g39480.1 // Bradi3g56070.1 // Bradi2g49420.1 // Bradi4g19060.1 // Bradi1g17270.1 // Bradi2g16290.1 // Bradi5g16440.1 // Bradi4g07930.1 // Bradi5g16590.1 // Bradi4g35780.1 // Bradi5g22830.1 // Bradi2g02510.1 // Bradi1g63600.1 // Bradi1g22650.1 // Bradi5g23680.1 // Bradi2g52380.1 // Bradi2g59490.1 // Bradi2g47220.1 // Bradi3g37600.1 // Bradi1g73720.1 // Bradi3g48880.1 // Bradi2g23520.1 // Bradi1g61170.1 // Bradi4g44760.1 // Bradi4g31270.1 // Bradi1g51540.1 // Bradi3g18580.1 // Bradi5g23890.1 // Bradi3g08660.1 // Bradi4g21980.1*

*GO:0006629 P lipid metabolic process*

*8 95 556 25219 0.0013 0.026*

*Bradi1g61170.1 // Bradi4g29600.1 // Bradi5g23680.1 // Bradi1g18490.1 // Bradi3g57100.1 // Bradi3g56070.1 // Bradi1g48870.1 // Bradi1g49020.1*

*GO:0006464 P protein modification process*

*17 95 1973 25219 0.0011 0.026*

*Bradi1g61170.1 // Bradi1g22650.1 // Bradi1g48880.1 // Bradi2g36470.1 // Bradi4g25880.1 // Bradi2g51560.1 // Bradi1g60170.1 // Bradi2g48830.1 // Bradi4g07930.1 // Bradi5g23890.1 // Bradi4g44460.1 // Bradi3g08660.1 // Bradi5g16590.1 // Bradi3g37600.1 // Bradi4g21980.1 // Bradi1g17270.1 // Bradi2g02510.1*

*GO:0043412 P macromolecule modification*

*17 95 2030 25219 0.0015 0.026*

*Bradi1g61170.1 // Bradi1g22650.1 // Bradi1g48880.1 // Bradi2g36470.1 // Bradi4g25880.1 // Bradi2g51560.1 // Bradi1g60170.1 // Bradi2g48830.1 // Bradi4g07930.1 // Bradi5g23890.1 // Bradi4g44460.1 // Bradi3g08660.1 // Bradi5g16590.1 // Bradi3g37600.1 // Bradi4g21980.1 // Bradi1g17270.1 // Bradi2g02510.1*

*GO:0044249 P cellular biosynthetic process*

*21 95 2879 25219 0.0022 0.029*

*Bradi4g39480.1 // Bradi1g63600.1 // Bradi2g23520.1 // Bradi2g52330.1 // Bradi1g61170.1 // Bradi1g48880.1 // Bradi3g18580.1 // Bradi5g23680.1 // Bradi2g52380.1 // Bradi2g49420.1 // Bradi4g19060.1 // Bradi2g47220.1 // Bradi1g73720.1 // Bradi3g48880.1 // Bradi1g72180.1 // Bradi2g59490.1 // Bradi4g45010.1 // Bradi2g50140.1 // Bradi4g35780.1 // Bradi4g35910.1 // Bradi1g21420.1*

*GO:0006811 P ion transport*

*7 95 461 25219 0.0019 0.029*

*Bradi1g74680.1 // Bradi2g33110.1 // Bradi3g17900.1 // Bradi5g21580.1 // Bradi1g53680.1 // Bradi2g22530.1 // Bradi2g22520.1*

*GO:0019538 P protein metabolic process*

*24 95 3447 25219 0.0019 0.029*

*Bradi2g52330.1 // Bradi1g48880.1 // Bradi2g36470.1 // Bradi4g25880.1 // Bradi3g37600.1 // Bradi2g48830.1 // Bradi1g45160.1 // Bradi4g44460.1 // Bradi1g17270.1 // Bradi3g44640.1 // Bradi2g51560.1 // Bradi5g16590.1 // Bradi4g07930.1 // Bradi2g02510.1 // Bradi1g22650.1 // Bradi4g13280.1 // Bradi3g21550.1 // Bradi1g60170.1 // Bradi1g73720.1 // Bradi1g61170.1 // Bradi2g46560.1 // Bradi5g23890.1 // Bradi3g08660.1 // Bradi4g21980.1*

*GO:0006796 P phosphate metabolic process*

*15 95 1746 25219 0.0023 0.029*

*Bradi1g22650.1 // Bradi2g36470.1 // Bradi4g25880.1 // Bradi2g51560.1 // Bradi1g60170.1 // Bradi2g48830.1 // Bradi4g07930.1 // Bradi5g23890.1 // Bradi4g44460.1 // Bradi3g08660.1 // Bradi5g16590.1 // Bradi3g37600.1 // Bradi4g21980.1 // Bradi1g17270.1 // Bradi2g02510.1*

*GO:0006793 P phosphorus metabolic process*

*15 95 1746 25219 0.0023 0.029*

*Bradi1g22650.1 // Bradi2g36470.1 // Bradi4g25880.1 // Bradi2g51560.1 // Bradi1g60170.1 // Bradi2g48830.1 // Bradi4g07930.1 // Bradi5g23890.1 // Bradi4g44460.1 // Bradi3g08660.1 // Bradi5g16590.1 // Bradi3g37600.1 // Bradi4g21980.1 // Bradi1g17270.1 // Bradi2g02510.1*

*GO:0043687 P post-translational protein modification*

*15 95 1769 25219 0.0026 0.031*

*Bradi1g22650.1 // Bradi2g36470.1 // Bradi4g25880.1 // Bradi2g51560.1 // Bradi1g60170.1 // Bradi2g48830.1 // Bradi4g07930.1 // Bradi5g23890.1 // Bradi4g44460.1 // Bradi3g08660.1 // Bradi5g16590.1 // Bradi3g37600.1 // Bradi4g21980.1 // Bradi1g17270.1 // Bradi2g02510.1*

GO:0009058 P biosynthetic process

21 95 3126 25219 0.0058 0.065 //

Bradi4g39480.1 // Bradi1g63600.1 // Bradi2g23520.1 // Bradi2g52330.1 // Bradi1g61170.1 // Bradi1g48880.1 // Bradi3g18580.1 // Bradi5g23680.1 // Bradi2g52380.1 // Bradi2g49420.1 // Bradi4g19060.1 // Bradi2g47220.1 // Bradi1g73720.1 // Bradi3g48880.1 // Bradi1g72180.1 // Bradi2g59490.1 // Bradi4g45010.1 // Bradi2g50140.1 // Bradi4g35780.1 // Bradi4g35910.1 // Bradi1g21420.1

GO:0034645 P cellular macromolecule biosynthetic process

16 95 2213 25219 0.0086 0.092

Bradi4g39480.1 // Bradi1g63600.1 // Bradi2g52330.1 // Bradi1g48880.1 // Bradi3g18580.1 // Bradi2g52380.1 // Bradi2g49420.1 // Bradi4g19060.1 // Bradi2g47220.1 // Bradi1g73720.1 // Bradi3g48880.1 // Bradi1g61170.1 // Bradi2g50140.1 // Bradi4g35780.1 // Bradi4g35910.1 // Bradi2g23520.1

GO:0009059 P macromolecule biosynthetic process

16 95 2228 25219 0.0091 0.093

Bradi4g39480.1 // Bradi1g63600.1 // Bradi2g52330.1 // Bradi1g48880.1 // Bradi3g18580.1 // Bradi2g52380.1 // Bradi2g49420.1 // Bradi4g19060.1 // Bradi2g47220.1 // Bradi1g73720.1 // Bradi3g48880.1 // Bradi1g61170.1 // Bradi2g50140.1 // Bradi4g35780.1 // Bradi4g35910.1 // Bradi2g23520.1

GO:0044267 P cellular protein metabolic process

19 95 2880 25219 0.011 0.1

Bradi1g61170.1 // Bradi2g52330.1 // Bradi1g22650.1 // Bradi1g48880.1 // Bradi2g36470.1 // Bradi4g25880.1 // Bradi2g51560.1 // Bradi1g60170.1 // Bradi2g48830.1 // Bradi4g07930.1 // Bradi5g23890.1 // Bradi4g44460.1 // Bradi3g08660.1 // Bradi5g16590.1 // Bradi3g37600.1 // Bradi4g21980.1 // Bradi1g17270.1 // Bradi1g73720.1 // Bradi2g02510.1

GO:0045449 P regulation of transcription

10 95 1214 25219 0.016 0.14

Bradi1g63600.1 // Bradi3g18580.1 // Bradi2g52380.1 // Bradi2g49420.1 // Bradi4g19060.1 // Bradi2g47220.1 // Bradi3g48880.1 // Bradi4g35910.1 // Bradi4g35780.1 // Bradi2g23520.1

GO:0031326 P regulation of cellular biosynthetic process

10 95 1227 25219 0.018 0.14

Bradi1g63600.1 // Bradi3g18580.1 // Bradi2g52380.1 // Bradi2g49420.1 // Bradi4g19060.1 // Bradi2g47220.1 // Bradi3g48880.1 // Bradi4g35910.1 // Bradi4g35780.1 // Bradi2g23520.1

GO:0019219 P regulation of nucleobase, nucleoside, nucleotide and nucleic acid metabolic process

10 95 1223 25219 0.017 0.14

Bradi1g63600.1 // Bradi3g18580.1 // Bradi2g52380.1 // Bradi2g49420.1 // Bradi4g19060.1 // Bradi2g47220.1 // Bradi3g48880.1 // Bradi4g35910.1 // Bradi4g35780.1 // Bradi2g23520.1

GO:0009889 P regulation of biosynthetic process

10 95 1227 25219 0.018 0.14

Bradi1g63600.1 // Bradi3g18580.1 // Bradi2g52380.1 // Bradi2g49420.1 // Bradi4g19060.1 // Bradi2g47220.1 // Bradi3g48880.1 // Bradi4g35910.1 // Bradi4g35780.1 // Bradi2g23520.1

GO:0050794 P regulation of cellular process

12 95 1604 25219 0.018 0.14 // Bradi1g63600.1 // Bradi3g18580.1 // Bradi4g25880.1 // Bradi2g52380.1 // Bradi2g49420.1 // Bradi4g19060.1 // Bradi2g47220.1 // Bradi3g48880.1 // Bradi4g44460.1 // Bradi4g35910.1 // Bradi4g35780.1 // Bradi2g23520.1

GO:0010556 P regulation of macromolecule biosynthetic process

10 95 1227 25219 0.018 0.14

Bradi1g63600.1 // Bradi3g18580.1 // Bradi2g52380.1 // Bradi2g49420.1 // Bradi4g19060.1 // Bradi2g47220.1 // Bradi3g48880.1 // Bradi4g35910.1 // Bradi4g35780.1 // Bradi2g23520.1

GO:0010468 P regulation of gene expression

10 95 1237 25219 0.019 0.14

Bradi1g63600.1 // Bradi3g18580.1 // Bradi2g52380.1 // Bradi2g49420.1 // Bradi4g19060.1 // Bradi2g47220.1 // Bradi3g48880.1 // Bradi4g35910.1 // Bradi4g35780.1 // Bradi2g23520.1

GO:0051171 P regulation of nitrogen compound metabolic process

10 95 1246 25219 0.019 0.14

Bradi1g63600.1 // Bradi3g18580.1 // Bradi2g52380.1 // Bradi2g49420.1 // Bradi4g19060.1 // Bradi2g47220.1 // Bradi3g48880.1 // Bradi4g35910.1 // Bradi4g35780.1 // Bradi2g23520.1

GO:0006807 P nitrogen compound metabolic process

17 95 2649 25219 0.02 0.14

Bradi4g39480.1 // Bradi4g44760.1 // Bradi3g18580.1 // Bradi1g63600.1 // Bradi5g04070.1 // Bradi2g52380.1 // Bradi2g49420.1 // Bradi4g19060.1 // Bradi2g47220.1 // Bradi3g48880.1 // Bradi3g48230.1 // Bradi1g72180.1 // Bradi2g59490.1 // Bradi4g45010.1 // Bradi4g35910.1 // Bradi4g35780.1 // Bradi2g23520.1

GO:0031323 P regulation of cellular metabolic process

10 95 1262 25219 0.021 0.14

Bradi1g63600.1 // Bradi3g18580.1 // Bradi2g52380.1 // Bradi2g49420.1 // Bradi4g19060.1 // Bradi2g47220.1 // Bradi3g48880.1 // Bradi4g35910.1 // Bradi4g35780.1 // Bradi2g23520.1

GO:0006810 P transport

11 95 1483 25219 0.024 0.16 //

Bradi1g74680.1 // Bradi2g33110.1 // Bradi4g31270.1 // Bradi4g23380.1 // Bradi2g22530.1 // Bradi1g37100.1 // Bradi1g53680.1 // Bradi2g22520.1 // Bradi1g71370.1 // Bradi3g17900.1 // Bradi5g21580.1

GO:0051234 P establishment of localization

11 95 1483 25219 0.024 0.16

Bradi1g74680.1 // Bradi2g33110.1 // Bradi4g31270.1 // Bradi4g23380.1 // Bradi2g22530.1 // Bradi1g37100.1 // Bradi1g53680.1 // Bradi2g22520.1 // Bradi1g71370.1 // Bradi3g17900.1 // Bradi5g21580.1

GO:0051179 P localization

11 95 1505 25219 0.027 0.17

Bradi1g74680.1 // Bradi2g33110.1 // Bradi4g31270.1 // Bradi4g23380.1 // Bradi2g22530.1 // Bradi1g37100.1 // Bradi1g53680.1 // Bradi2g22520.1 // Bradi1g71370.1 // Bradi3g17900.1 // Bradi5g21580.1

GO:0080090 P regulation of primary metabolic process

10 95 1326 25219 0.028 0.17

Bradi1g63600.1 // Bradi3g18580.1 // Bradi2g52380.1 // Bradi2g49420.1 // Bradi4g19060.1 // Bradi2g47220.1 // Bradi3g48880.1 // Bradi4g35910.1 // Bradi4g35780.1 // Bradi2g23520.1

GO:0050789 P regulation of biological process

12 95 1737 25219 0.03 0.18

Bradi1g63600.1 // Bradi3g18580.1 // Bradi4g25880.1 // Bradi2g52380.1 // Bradi2g49420.1 // Bradi4g19060.1 // Bradi2g47220.1 // Bradi3g48880.1 // Bradi4g44460.1 // Bradi4g35910.1 // Bradi4g35780.1 // Bradi2g23520.1

GO:0060255 P regulation of macromolecule metabolic process

10 95 1343 25219 0.03 0.18

Bradi1g63600.1 // Bradi3g18580.1 // Bradi2g52380.1 // Bradi2g49420.1 // Bradi4g19060.1 // Bradi2g47220.1 // Bradi3g48880.1 // Bradi4g35910.1 // Bradi4g35780.1 // Bradi2g23520.1

GO:0006350 P transcription

10 95 1370 25219 0.034 0.19

Bradi1g63600.1 // Bradi3g18580.1 // Bradi2g52380.1 // Bradi2g49420.1 // Bradi4g19060.1 // Bradi2g47220.1 // Bradi3g48880.1 // Bradi4g35910.1 // Bradi4g35780.1 // Bradi2g23520.1

GO:0019222 P regulation of metabolic process

10 95 1395 25219 0.038 0.21

Bradi1g63600.1 // Bradi3g18580.1 // Bradi2g52380.1 // Bradi2g49420.1 // Bradi4g19060.1 // Bradi2g47220.1 //

Bradi3g48880.1 // Bradi4g35910.1 // Bradi4g35780.1 // Bradi2g23520.1

GO:0065007 P biological regulation

12 95 1833 25219 0.043 0.23

Bradi1g63600.1 // Bradi3g18580.1 // Bradi4g25880.1 // Bradi2g52380.1 // Bradi2g49420.1 // Bradi4g19060.1 // Bradi2g47220.1 // Bradi3g48880.1 // Bradi4g44460.1 // Bradi4g35910.1 // Bradi4g35780.1 // Bradi2g23520.1

**Functional Processes**

**GO:0046873 F metal ion transmembrane transporter activity**

**6 95 91 25219 1.8e-06 0.0003**

**Bradi2g33110.1 // Bradi3g17900.1 // Bradi5g21580.1 // Bradi1g53680.1 // Bradi2g22530.1 // Bradi2g22520.1**

**GO:0016740 F transferase activity**

**28 95 3270 25219 1.9e-05 0.0011**

**Bradi1g48880.1 // Bradi2g36470.1 // Bradi4g25880.1 // Bradi3g37600.1 // Bradi2g48830.1 // Bradi4g44460.1 // Bradi5g24290.1 // Bradi2g50140.1 // Bradi1g17270.1 // Bradi4g39480.1 // Bradi3g56070.1 // Bradi2g51560.1 // Bradi5g16590.1 // Bradi5g16120.1 // Bradi4g07930.1 // Bradi2g02510.1 // Bradi1g22650.1 // Bradi5g23680.1 // Bradi3g11300.1 // Bradi1g60170.1 // Bradi4g12190.1 // Bradi1g61170.1 // Bradi4g27360.1 // Bradi5g23890.1 // Bradi3g08660.1 // Bradi3g33080.1 // Bradi4g21980.1 // Bradi2g36910.1**

**GO:0003824 F catalytic activity**

**55 95 9170 25219 1.7e-05 0.0011**

**Bradi4g29600.1 // Bradi1g48880.1 // Bradi3g06780.1 // Bradi2g36910.1 // Bradi4g25880.1 // Bradi1g18490.1 // Bradi3g57100.1 // Bradi2g48830.1 // Bradi1g45160.1 // Bradi4g44460.1 // Bradi1g48870.1 // Bradi1g72180.1 // Bradi4g45010.1 // Bradi4g23380.1 // Bradi2g50140.1 // Bradi1g17270.1 // Bradi5g24650.1 // Bradi4g39480.1 // Bradi3g56070.1 // Bradi2g36470.1 // Bradi2g24090.1 // Bradi1g04590.1 // Bradi2g51560.1 // Bradi5g23680.1 // Bradi5g16590.1 // Bradi1g21420.1 // Bradi3g16460.1 // Bradi4g07930.1 // Bradi5g22830.1 // Bradi2g02510.1 // Bradi5g23890.1 // Bradi2g44850.1 // Bradi1g22650.1 // Bradi5g04070.1 // Bradi5g16120.1 // Bradi3g04750.1 // Bradi3g11300.1 // Bradi3g21550.1 // Bradi3g37600.1 // Bradi1g49020.1 // Bradi4g12190.1 // Bradi1g61170.1 // Bradi4g44760.1 // Bradi1g51540.1 // Bradi1g06030.1 // Bradi2g46560.1 // Bradi4g13280.1 // Bradi4g27360.1 // Bradi5g24290.1 // Bradi3g08660.1 // Bradi3g48230.1 // Bradi3g33080.1 // Bradi4g21980.1 // Bradi1g60170.1 // Bradi5g16440.1**

**GO:0005488 F binding**

**52 95 8851 25219 7.3e-05 0.003**

**Bradi1g74680.1 // Bradi1g63600.1 // Bradi2g52330.1 // Bradi3g06780.1 // Bradi4g25880.1 // Bradi1g60170.1 // Bradi2g48830.1 // Bradi1g45160.1 // Bradi4g44460.1 // Bradi4g23380.1 // Bradi4g35910.1 // Bradi1g17270.1 // Bradi5g24650.1 // Bradi4g39480.1 // Bradi2g36470.1 // Bradi3g44640.1 // Bradi4g40260.1 // Bradi2g51560.1 // Bradi4g19060.1 // Bradi1g37100.1 // Bradi5g16590.1 // Bradi4g13600.1 // Bradi3g16460.1 // Bradi4g07930.1 // Bradi5g18460.1 // Bradi4g35780.1 // Bradi5g22830.1 // Bradi4g40250.1 // Bradi2g02510.1 // Bradi5g09510.1 // Bradi2g44850.1 // Bradi1g22650.1 // Bradi5g14280.1 // Bradi5g04070.1 // Bradi2g52380.1 // Bradi2g59490.1 // Bradi1g13690.1 // Bradi2g47220.1 // Bradi3g54460.1 // Bradi3g37600.1 // Bradi3g04750.1 // Bradi2g18650.1 // Bradi4g44760.1 // Bradi1g54800.1 // Bradi1g06030.1 // Bradi3g18580.1 // Bradi1g68300.1 // Bradi5g23890.1 // Bradi3g08660.1 // Bradi3g48230.1 // Bradi4g21980.1 // Bradi2g25580.1**

*GO:0004713 F protein tyrosine kinase activity*

*10 95 797 25219 0.0009 0.017*

*Bradi1g22650.1 // Bradi4g07930.1 // Bradi2g51560.1 // Bradi5g23890.1 // Bradi3g08660.1 // Bradi1g60170.1 // Bradi4g21980.1 // Bradi3g37600.1 // Bradi1g17270.1 // Bradi2g02510.1*

*GO:0016301 F kinase activity*

*16 95 1756 25219 0.00088 0.017*

*Bradi1g22650.1 // Bradi2g36470.1 // Bradi3g56070.1 // Bradi4g25880.1 // Bradi2g51560.1 // Bradi1g60170.1 // Bradi2g48830.1 // Bradi4g07930.1 // Bradi5g23890.1 // Bradi4g44460.1 // Bradi3g08660.1 // Bradi5g16590.1 // Bradi3g37600.1 // Bradi4g21980.1 // Bradi1g17270.1 // Bradi2g02510.1*

*GO:0016773 F phosphotransferase activity, alcohol group as acceptor*

*16 95 1714 25219 0.00068 0.017*

*Bradi1g22650.1 // Bradi2g36470.1 // Bradi3g56070.1 // Bradi4g25880.1 // Bradi2g51560.1 // Bradi1g60170.1 // Bradi2g48830.1 // Bradi4g07930.1 // Bradi5g23890.1 // Bradi4g44460.1 // Bradi3g08660.1 // Bradi5g16590.1 // Bradi3g37600.1 // Bradi4g21980.1 // Bradi1g17270.1 // Bradi2g02510.1*

*GO:0004672 F protein kinase activity*

*15 95 1566 25219 0.00078 0.017*

*Bradi1g22650.1 // Bradi2g36470.1 // Bradi4g25880.1 // Bradi2g51560.1 // Bradi1g60170.1 // Bradi2g48830.1 // Bradi4g07930.1 // Bradi5g23890.1 // Bradi4g44460.1 // Bradi3g08660.1 // Bradi5g16590.1 // Bradi3g37600.1 // Bradi4g21980.1 // Bradi1g17270.1 // Bradi2g02510.1*

*GO:0008324 F cation transmembrane transporter activity*

*6 95 266 25219 0.00057 0.017*

*Bradi2g33110.1 // Bradi3g17900.1 // Bradi5g21580.1 // Bradi1g53680.1 // Bradi2g22530.1 // Bradi2g22520.1*

*GO:0016772 F transferase activity, transferring phosphorus-containing groups*

*17 95 2043 25219 0.0016 0.027*

*Bradi4g39480.1 // Bradi1g22650.1 // Bradi2g36470.1 // Bradi3g56070.1 // Bradi4g25880.1 // Bradi2g51560.1 // Bradi1g60170.1 // Bradi2g48830.1 // Bradi4g07930.1 // Bradi5g23890.1 // Bradi4g44460.1 // Bradi3g08660.1 // Bradi5g16590.1 // Bradi3g37600.1 // Bradi4g21980.1 // Bradi1g17270.1 // Bradi2g02510.1*

*GO:0016757 F transferase activity, transferring glycosyl groups*

*7 95 491 25219 0.0027 0.039*

*Bradi1g48880.1 // Bradi5g24290.1 // Bradi3g11300.1 // Bradi4g27360.1 // Bradi5g16120.1 // Bradi2g50140.1 // Bradi3g33080.1*

*GO:0017076 F purine nucleotide binding*

*23 95 3432 25219 0.0039 0.039*

*Bradi2g36470.1 // Bradi4g25880.1 // Bradi3g37600.1 // Bradi2g48830.1 // Bradi1g45160.1 // Bradi4g44460.1 // Bradi4g23380.1 // Bradi1g17270.1 // Bradi3g44640.1 // Bradi4g40260.1 // Bradi2g51560.1 // Bradi5g16590.1 // Bradi3g16460.1 // Bradi4g07930.1 // Bradi2g02510.1 // Bradi1g22650.1 // Bradi1g13690.1 // Bradi3g04750.1 // Bradi1g60170.1 // Bradi1g54800.1 // Bradi5g23890.1 // Bradi3g08660.1 // Bradi4g21980.1*

*GO:0005524 F ATP binding*

*21 95 3063 25219 0.0046 0.039*

*Bradi1g22650.1 // Bradi3g44640.1 // Bradi2g36470.1 // Bradi5g23890.1 // Bradi4g40260.1 // Bradi4g25880.1 // Bradi2g51560.1 // Bradi4g23380.1 // Bradi1g60170.1 // Bradi2g48830.1 // Bradi4g07930.1 // Bradi1g45160.1 // Bradi4g44460.1 // Bradi3g08660.1 // Bradi5g16590.1 // Bradi3g37600.1 // Bradi4g21980.1 // Bradi1g54800.1 // Bradi1g17270.1 // Bradi3g04750.1 // Bradi2g02510.1*

*GO:0015075 F ion transmembrane transporter activity*

*6 95 380 25219 0.0034 0.039*

*Bradi2g33110.1 // Bradi3g17900.1 // Bradi5g21580.1 // Bradi1g53680.1 // Bradi2g22530.1 // Bradi2g22520.1*

*GO:0032559 F adenyl ribonucleotide binding*

*21 95 3064 25219 0.0046 0.039*

*Bradi1g22650.1 // Bradi3g44640.1 // Bradi2g36470.1 // Bradi5g23890.1 // Bradi4g40260.1 // Bradi4g25880.1 // Bradi2g51560.1 // Bradi4g23380.1 // Bradi1g60170.1 // Bradi2g48830.1 // Bradi4g07930.1 // Bradi1g45160.1 // Bradi4g44460.1 // Bradi3g08660.1 // Bradi5g16590.1 // Bradi3g37600.1 // Bradi4g21980.1 // Bradi1g54800.1 // Bradi1g17270.1 // Bradi3g04750.1 // Bradi2g02510.1*

*GO:0032555 F purine ribonucleotide binding*

*22 95 3275 25219 0.0047 0.039*

*Bradi2g36470.1 // Bradi4g25880.1 // Bradi3g37600.1 // Bradi2g48830.1 // Bradi1g45160.1 // Bradi4g44460.1 // Bradi1g22650.1 // Bradi1g17270.1 // Bradi3g44640.1 // Bradi4g40260.1 // Bradi2g51560.1 // Bradi5g16590.1 // Bradi4g07930.1 // Bradi2g02510.1 // Bradi4g23380.1 // Bradi1g13690.1 // Bradi3g04750.1 // Bradi1g60170.1 // Bradi1g54800.1 // Bradi5g23890.1 // Bradi3g08660.1 // Bradi4g21980.1*

*GO:0032553 F ribonucleotide binding*

*22 95 3275 25219 0.0047 0.039*

*Bradi2g36470.1 // Bradi4g25880.1 // Bradi3g37600.1 // Bradi2g48830.1 // Bradi1g45160.1 // Bradi4g44460.1 // Bradi1g22650.1 // Bradi1g17270.1 // Bradi3g44640.1 // Bradi4g40260.1 // Bradi2g51560.1 // Bradi5g16590.1 // Bradi4g07930.1 // Bradi2g02510.1 // Bradi4g23380.1 // Bradi1g13690.1 // Bradi3g04750.1 // Bradi1g60170.1 // Bradi1g54800.1 // Bradi5g23890.1 // Bradi3g08660.1 // Bradi4g21980.1*

*GO:0022891 F substrate-specific transmembrane transporter activity*

*6 95 411 25219 0.0049 0.039*

*Bradi2g33110.1 // Bradi3g17900.1 // Bradi5g21580.1 // Bradi1g53680.1 // Bradi2g22530.1 // Bradi2g22520.1*

*GO:0030554 F adenyl nucleotide binding*

*22 95 3215 25219 0.0038 0.039*

*Bradi2g36470.1 // Bradi4g25880.1 // Bradi3g37600.1 // Bradi2g48830.1 // Bradi1g45160.1 // Bradi4g44460.1 // Bradi4g23380.1 // Bradi1g17270.1 // Bradi3g44640.1 // Bradi4g40260.1 // Bradi2g51560.1 // Bradi5g16590.1 // Bradi3g16460.1 // Bradi4g07930.1 // Bradi2g02510.1 // Bradi1g22650.1 // Bradi3g04750.1 // Bradi1g60170.1 // Bradi1g54800.1 // Bradi5g23890.1 // Bradi3g08660.1 // Bradi4g21980.1*

*GO:0001883 F purine nucleoside binding*

*22 95 3215 25219 0.0038 0.039*

*Bradi2g36470.1 // Bradi4g25880.1 // Bradi3g37600.1 // Bradi2g48830.1 // Bradi1g45160.1 // Bradi4g44460.1 // Bradi4g23380.1 // Bradi1g17270.1 // Bradi3g44640.1 // Bradi4g40260.1 // Bradi2g51560.1 // Bradi5g16590.1 // Bradi3g16460.1 // Bradi4g07930.1 // Bradi2g02510.1 // Bradi1g22650.1 // Bradi3g04750.1 // Bradi1g60170.1 // Bradi1g54800.1 // Bradi5g23890.1 // Bradi3g08660.1 // Bradi4g21980.1*

*GO:0001882 F nucleoside binding*

*22 95 3216 25219 0.0038 0.039*

*Bradi2g36470.1 // Bradi4g25880.1 // Bradi3g37600.1 // Bradi2g48830.1 // Bradi1g45160.1 // Bradi4g44460.1 // Bradi4g23380.1 // Bradi1g17270.1 // Bradi3g44640.1 // Bradi4g40260.1 // Bradi2g51560.1 // Bradi5g16590.1 // Bradi3g16460.1 // Bradi4g07930.1 // Bradi2g02510.1 // Bradi1g22650.1 // Bradi3g04750.1 // Bradi1g60170.1 // Bradi1g54800.1 // Bradi5g23890.1 // Bradi3g08660.1 // Bradi4g21980.1*

*GO:0000166 F nucleotide binding*

*23 95 3557 25219 0.006 0.046*

*Bradi2g36470.1 // Bradi4g25880.1 // Bradi3g37600.1 // Bradi2g48830.1 // Bradi1g45160.1 // Bradi4g44460.1 // Bradi4g23380.1 // Bradi1g17270.1 // Bradi3g44640.1 // Bradi4g40260.1 // Bradi2g51560.1 // Bradi5g16590.1 // Bradi3g16460.1 // Bradi4g07930.1 // Bradi2g02510.1 // Bradi1g22650.1 // Bradi1g13690.1 // Bradi3g04750.1 // Bradi1g60170.1 // Bradi1g54800.1 // Bradi5g23890.1 // Bradi3g08660.1 // Bradi4g21980.1*

GO:0016788 F hydrolase activity, acting on ester bonds

7 95 627 25219 0.0099 0.071

Bradi4g29600.1 // Bradi1g18490.1 // Bradi3g57100.1 // Bradi3g56070.1 // Bradi1g48870.1 // Bradi2g24090.1 // Bradi1g49020.1

GO:0022892 F substrate-specific transporter activity

6 95 492 25219 0.011 0.078

Bradi2g33110.1 // Bradi3g17900.1 // Bradi5g21580.1 // Bradi1g53680.1 // Bradi2g22530.1 // Bradi2g22520.1

GO:0022857 F transmembrane transporter activity

6 95 590 25219 0.025 0.16

Bradi2g33110.1 // Bradi3g17900.1 // Bradi5g21580.1 // Bradi1g53680.1 // Bradi2g22530.1 // Bradi2g22520.1

GO:0030528 F transcription regulator activity

8 95 981 25219 0.032 0.21

Bradi3g48880.1 // Bradi2g52380.1 // Bradi3g18580.1 // Bradi4g19060.1 // Bradi2g47220.1 // Bradi4g35910.1 // Bradi4g35780.1 // Bradi2g23520.1

GO:0003700 F transcription factor activity

6 95 641 25219 0.035 0.22

Bradi2g52380.1 // Bradi3g18580.1 // Bradi4g19060.1 // Bradi2g47220.1 // Bradi4g35910.1 // Bradi4g35780.1

GO:0020037 F heme binding

5 95 521 25219 0.048 0.29

Bradi2g44850.1 // Bradi1g06030.1 // Bradi5g24650.1 // Bradi3g06780.1 // Bradi3g04750.1
